# Tumor Immune Microenvironment in Gynecologic Cancers

**DOI:** 10.3390/cancers15153849

**Published:** 2023-07-28

**Authors:** Daniel Margul, Camilla Yu, Mariam M. AlHilli

**Affiliations:** Department of Obstetrics and Gynecology, Division of Gynecologic Oncology, Cleveland Clinic, Cleveland, OH 44195, USA; marguld@ccf.org (D.M.); yuc2@ccf.org (C.Y.)

**Keywords:** gynecologic oncology, ovarian cancer, endometrial cancer, cervical cancer, vaginal cancer, vulvar cancer, tumor immune microenvironment, tumor infiltrating lymphocytes, pembrolizumab, checkpoint inhibitors

## Abstract

**Simple Summary:**

Gynecologic cancers represent a broad spectrum of diseases within the female genital tract. In recent years, the tumor microenvironment has become an active area of investigation leading to the development of novel therapeutics targeting the immune system and shifts in traditional treatment paradigms for gynecologic malignancies. In this review, we delineate what is known currently regarding the tumor immune microenvironment (TIME) in ovarian, endometrial, cervical, and vaginal/vulvar cancers. Additionally, we will review clinical trial data to demonstrate how we are leveraging what we know about the TIME to treat gynecologic malignancies.

**Abstract:**

Gynecologic cancers have varying response rates to immunotherapy due to the heterogeneity of each cancer’s molecular biology and features of the tumor immune microenvironment (TIME). This article reviews key features of the TIME and its role in the pathophysiology and treatment of ovarian, endometrial, cervical, vulvar, and vaginal cancer. Knowledge of the role of the TIME in gynecologic cancers has been rapidly developing with a large body of preclinical studies demonstrating an intricate yet dichotomous role that the immune system plays in either supporting the growth of cancer or opposing it and facilitating effective treatment. Many targets and therapeutics have been identified including cytokines, antibodies, small molecules, vaccines, adoptive cell therapy, and bacterial-based therapies but most efforts in gynecologic cancers to utilize them have not been effective. However, with the development of immune checkpoint inhibitors, we have started to see the rapid and successful employment of therapeutics in cervical and endometrial cancer. There remain many challenges in utilizing the TIME, particularly in ovarian cancer, and further studies are needed to identify and validate efficacious therapeutics.

## 1. Introduction

Our understanding of the interplay of immune cell populations and tumor cells within the TIME has grown substantially in recent years. Research has unveiled the distribution, composition, and function of immune cells within tumors and the association between immune cell populations and prognosis. The extent and characteristics of immune cell populations within the TIME correlates with the disease extent and treatment response. 

Understanding of the TIME has also led to the development of numerous therapeutic targets that seek to enhance the immune system’s capacity to eliminate or prevent the progression of cancer, collectively referred to as “immunotherapy”. These therapies include immune checkpoint therapy, adoptive cell therapy, cytokine therapy, monoclonal antibodies, and cancer vaccines. Immunotherapy has shifted the treatment paradigm for many solid malignancies including melanoma, non-small cell lung cancer, and gynecologic malignancies. In gynecologic cancers, approved therapies are limited to immune checkpoint inhibitors; however, the field is rapidly advancing with many putative therapeutics being investigated in ongoing clinical trials. The objectives of this review are three-fold:

(1) Provide an overview of what is known regarding the TIME in ovarian cancer, endometrial cancer, cervical cancer, vulvar cancer, and vaginal cancer;
(2) Review current literature regarding the association of certain immune subpopulations with treatment response and prognosis;
(3) Summarize key advances and the current landscape of immunotherapy in the treatment of gynecologic malignancies.

## 2. Ovarian Cancer

Ovarian cancer is the second most common gynecologic malignancy in the United States with an estimated 19,880 new cases and 12,810 new deaths in 2022 [1]. In 2020, it was estimated that the worldwide incidence was 313,959 cases with 207,252 deaths, making it the third most common and the second most deadly gynecologic cancer [2]. Due to a lack of efficacious screening and the absence of specific symptoms in early disease, patients typically present with advanced-stage disease. In addition to the widespread disease at presentation, the development of resistance to chemotherapy leads to recurrence in 70–90% of patients with limited treatment options [3]. Given the poor outcomes associated with advanced disease and treatment resistance, there is a significant need for the identification of novel treatment targets and therapeutics. In recent years, the TIME and the milieu of immune cells and cytokines within the tumor microenvironment have been identified to have a significant role in both tumorigenesis and chemotherapeutic resistance in ovarian cancer. 

### 2.1. Tumor Immune Microenvironment in Ovarian Cancer and Its Association with Outcomes

Macrophages act as key mediators of the immune system’s response to ovarian cancer as well as tumorigenesis. Macrophages residing in tumors, referred to as tumor-associated macrophages (TAMs), are classified into M1 and M2 macrophages [4]. When stimulated with interferon-gamma (IFN-ɣ), lipopolysaccharide (LPS), and granulocyte-macrophage-colony stimulating factor (GM-CSF), monocytes differentiate into M1 macrophages. M1 macrophages secrete IL-1, IL-12, TNFα, and CXCL12 and have cytotoxic activity, suppress tumor growth, and stimulate the immune system [5]. M2 macrophages, in contrast, develop when monocytes are stimulated with IL-4, IL-10, and IL-13 and suppress the immune system, thereby promoting tumor growth [6]. Increased numbers of M1 TAMs and higher M1/M2 ratios are both associated with longer overall survival (OS), whereas the higher density of CD163+ M2 TAMs is associated with worse progression-free survival (PFS) in epithelial ovarian cancers (EOCs) [7,8,9]. 

Neutrophils, like macrophages, exist on a spectrum of phenotypes. Like with macrophages, N1 and N2 neutrophils serve to either drive or suppress the immune system within the tumor immune microenvironment [10]. N1-type neutrophils are cytotoxic to tumor cells and express pro-inflammatory cytokines while N2-type neutrophils suppress the anti-tumor response [11]. Mechanistically, neutrophils release a milieu of growth factors, myeloperoxidases, reactive oxygen species, vascular endothelial growth factors, and matrix metalloproteinase-9 whose interplay is involved with tumorigenesis, growth, proliferation, and metastasis [10]. In ovarian cancer, neutrophils are also involved in establishing the pre-metastatic niche within the omentum via neutrophil extracellular traps [12]. While outcomes based on neutrophil phenotypes have not been identified, the neutrophil to lymphocyte ratio has been shown to be a surrogate for survival. In a meta-analysis by Chen et al., patients who had decreased neutrophil to lymphocyte ratios in their blood have higher overall survival (OS) (hazard ratio (HR) 1.4; 95% confidence interval (CI) 1.11–1.79, *p* = 0.005) and progression-free survival (PFS) (HR 1.52, 95% CI 1.19–1.96, *p* = 0.001) [13]. Neutrophils may also facilitate immune escape by T cell suppression and PD-L1 upregulation and, in accordance, the neutrophil/lymphocyte ratio correlates with increased Th17, IL-17, and PD-L1 levels [14].

Other cells, such as natural killer (NK) cells and dendritic cells, also have associations with treatment outcomes. Reports characterizing the predictive value of NK cells are mixed. One study demonstrated that the NK cell density of ≥10 cells/mm^2^ was associated with longer OS, increased from 29 months to 45 months (HR 0.67; *p* = 0.041) in patients with high-grade serous ovarian cancer (HGSOC) [15]. A second study had mixed results, suggesting that intraepithelial NK cells were more beneficial than stromal NK cells; a meta-analysis only demonstrated a trend to improved OS (HR 0.57; 95% CI, 0.26–1.24, *p* = 0.089) [16,17]. Dendritic cells serve an essential role in the TIME: they capture and present antigens to T cells, induce T cell activation and proliferation, and stimulate antitumor activity [18]. In two Czech cohorts of 81 and 66 patients with HGSOC, mature dendritic cells are associated with a Th1 immune response and favorable OS with an HR of 0.62 (95% CI, 0.44–0.87, *p* = 0.0057) in multivariate analysis [19]. However, immature dendritic cells may be associated with more tolerogenic states [20]. 

Cell populations of the adaptive immune response, such as T cells and their specific subtypes, are also predictive of ovarian cancer outcomes. Intratumoral CD3+ (helper and cytotoxic) T-cell infiltration is associated with both improved OS and delayed relapse [21]. CD8+ T cell infiltration has been associated with prolonged PFS and OS in several studies [22,23,24]. Regulatory T Cells (Treg), on the other hand, are associated with poor clinical outcomes with decreased survival linked to a tolerogenic state [25]. B cells have a less well-defined role with mixed results in the literature. CD138+ plasma cell tumor infiltration and CD19+ B cells in the omentum predict poor survival [26,27]. On the other hand, CD20+ B cells are predictive of positive survival, particularly when associated with CD8+ TILs [28,29,30].

In addition, there is extensive interpatient, intertumor, and intratumor heterogeneity as well as numerous histologic and immune subtypes that further increase the complexity of the TIME. Some groups have attempted to characterize this complexity by analyzing the transcriptome and by identifying molecular and/or immune subtypes of HGSOC. Olbrecht et al. used single cell RNA sequencing to provide transcriptomic resolution of these subtypes and correlated them with patient outcomes. Among 7 patients with HGSOC, they identified 11 cancer and 32 stromal phenotypes in HGSOC tumors. Of these, TGF-β driven fibroblasts, mesothelial cells, and lymphatic endothelial cells were predictors of poor outcomes while plasma cells correlated with favorable outcomes [31]. Another study by James et al. sought to elucidate chemotherapy-induced effects on immunotranscriptomics, cancer related pathways, and the relationship between the TIME and response to therapy. Neoadjuvant chemotherapy (NACT) increased pro-tumorigenic and immunoregulatory pathways as well as immune infiltration. In 31 HGSOC samples, pre-NACT immune cell populations did not correlate with platinum free interval (PFI). There were, however, relationships between PFI and post-NACT levels of exhausted CD8+T cells, NK cells, dendritic cells, and mast cells. Similarly, while no pathways were associated with PFI pre-NACT, post-NACT, Hedgehog, MAPK, Notch, PI3K, TGF-β, and Wnt signaling were all associated with longer PFI. In addition, they noted that ATF3 and AREG upregulation correlated with shorter PFI [32]. Lodewijk et al. investigated the mutational landscape and immune profiles of epithelial ovarian cancer samples at biopsy and interval debulking after NACT [33]. No major differences in the mutational landscape were identified pre- and post-NACT; however, the loss of heterozygosity was higher in patients with complete or near-complete responses. NACT was found to augment CD8+ T cells and for patients with a poor response to NACT, several immune regulatory pathways were activated, suggesting a possible role for immunotherapy. Another study found that NACT increased NK and T cell infiltration in a location-specific manner thought to be secondary to tumor heterogeneity [34]. Using HGSOC sequencing data from The Cancer Genome Atlas (TCGA) program and single-cell RNA sequencing from four patients, Wang et al. classified ovarian cancer tumors using a macrophage risk score based on M1/M2 macrophages and a gene risk score based on genes associated with the macrophage risk score. They were able to predict better OS and an inflammatory environment with their scoring systems and found that macrophage and CD8+ T cell signaling was a key feature. They created models for predicting prognosis and hub genes for potential checkpoint inhibitor responses [35]. Yuan et al. used unsupervised hierarchical clustering of transcriptomic data to identify three immunogenic subgroups: hyperimmunogenic, moderately immunogenic, and hypoimmunogenic. Hyperimmunogenic tumors were associated with improved survival and were found to be more likely to respond to immunotherapy [36]. Though the use of transcriptomics to classify patients has shown some promise in these retrospective datasets, leveraging the complexity of this data remains in its infancy and whether it can be used to augment therapy or patient outcomes remains to be determined. 

The TIME in ovarian cancer as well as other cancers is dichotomous, with the potential to create both an inflammatory environment with antitumoral activity that is associated with improved survival or to be immunosuppressive, creating an environment relatively favorable for cancer growth. Though recent work has revealed much of the role of the individual components of the TIME in ovarian cancer, the development of therapeutics is ongoing.

### 2.2. Therapeutic Targets in Ovarian Cancer

Though no pharmaceuticals have demonstrated clinical efficacy, TAMs are a potential target for ovarian cancer therapeutics and may be targeted through multiple mechanisms including the suppression of recruitment and shifting of polarization. M-CSF is a macrophage chemokine and may be targeted through its receptor, CSF-1R. CSF-1R inhibition in mouse studies has been shown to suppress proliferation and metastasis of ovarian cancer by acting through receptors directly on tumor cells, via TAM suppression, and by reduced M2 macrophage infiltration [37,38]. In another mouse model, reducing macrophage recruitment through the blockade of CSF-1R reduced metastases when given with docetaxel [39]. Likewise, selective inhibition of CSF-1R reduces ascites accumulation and M2 macrophage infiltration [37]. CCL2 is a chemokine for T cells, NK cells, and monocytes and is involved in metastasis through interactions with TAMs. In mice, a CCL2 inhibitor improved the efficacy of carboplatin and paclitaxel [40]. Additionally, in a phase I trial, in one patient with ovarian cancer, carlumab, an anti-CCL2 antibody, had a 50% reduction in CA-125 levels and 10.5 months of stable disease [41]. Unfortunately, no patients had an objective response and the drug was discontinued. 

Shifting the polarization balance away from M2 and towards M1 phenotypes would theoretically create a less tolerant and more tumoricidal environment. In a small phase I trial, 852A, a toll-like receptor (TLR) agonist, was used to stimulate NF-κB activation and the M1 phenotype; unfortunately, only one of nine patients with ovarian cancer achieved stable disease and treatment was associated with substantial toxicity [42]. IL-12 can similarly drive macrophages towards M1 polarization and has been shown to cause reduced tumor growth and even tumor regression in murine ovarian cancer models [43]. IL-12 plasmids were evaluated in both phase I and II trials [44]. In a phase II trial of IP EGEN-001 (IL-12 plasmid-based therapy) for platinum resistant ovarian cancer, 35% of patients had stable disease and 45% progressed [45]. Intraperitoneal IL-12 (GEN-1) has also been studied in combination with doxorubicin in patients with persistent or recurrent platinum resistance epithelial ovarian cancer, achieving partial responses in 21.4% of patients and stable disease in 35.7% [46]. OVATION2, a phase I/II study of GEN-1 with neoadjuvant chemotherapy, is ongoing (NCT03393884). STAT3, a member of the STAT (Signal Transducers and Activators of Transcription) family, can be modulated via a variety of molecular signals and has downstream effects on the cell cycle as well as macrophage polarization. In both in vitro and in vivo models, the STAT3 inhibitors, HO-3867 and WP1066, have been shown to induce apoptosis, cell cycle arrest, and chemosensitivity while decreasing tumor growth and metastasis [47,48]. Though promising in vitro, STAT3 signaling inhibition has only made it into a single trial. NCT02417753 investigated the STAT3 antisense oligonucleotide AZD9150 but the study was terminated due to difficulties with accrual.

Other studies have focused on activating the immune system as a whole rather than affecting individual cellular populations. MRx0518 is a strain of *Enterococcus gallinarum* that induces broad and robust activation of the immune system and thus anti-tumor activity mediated by toll-like receptor 5 (TLR-5) [49]. Ovarian cancer patients are included in an ongoing trial investigating MRX0518 in the preoperative stage prior to surgical resection (NCT03934827).

Though efforts to target the TIME in ovarian cancer in has seen promising preclinical results in both in vitro and in vivo models, the therapeutic efficacy was limited in clinical trials. Despite the limited success thus far, the importance of these mechanisms has been identified and they remain as putative therapeutic avenues. Further investigations are ongoing in several clinical trials and the outcomes are highly anticipated. 

### 2.3. Checkpoint Inhibitors in Ovarian Cancer

Immune checkpoint inhibitors have demonstrated limited efficacy in the setting of recurrent ovarian cancer. Several early-phase studies have investigated the efficacy of immune checkpoint inhibitors. The largest of these studies was Keynote-100, a phase II trial that included 376 patients with recurrent ovarian cancer, who were treated with pembrolizumab [50]. Cohort A received 1–3 prior lines of therapy with a platinum-free or treatment-free interval of 3–12 months and cohort B received 4–6 prior lines of therapy with a platinum-free or treatment-free interval of at least 3 months. The ORR was 7.5% in cohort A and 9% in cohort B, though when all patients were stratified by CPS, the ORR was 5.0% for CPS < 1, 10.2% for CPS ≥ 1, and 17.1% for CPS ≥ 10. The median OS was 17.6 for cohort B but not reached for cohort A.

CTLA-4, a transmembrane glycoprotein on T cells is well known to mediate the antitumor response. Nivolumab, a PD-1 monoclonal antibody, has been evaluated alone and with ipilimumab, a monoclonal antibody to CTLA-4 in a phase II study [51]. Compared to nivolumab alone, the combination had significantly improved, though still limited response rates (12.2% versus 31.4%) and PFS (2 versus 3.9 months). Of note, only a small number of patients were PD-L1 positive but the PD-L1 status was not associated with a response in either treatment group.

Javelin 100 was a phase III RCT investigating the use of avelumab, an anti-PD-L1 monoclonal antibody, in the frontline setting as a combination or maintenance therapy with carboplatin and paclitaxel [52]. Unfortunately, this trial was closed early due to insufficient efficacy. Javelin-200 was a phase III randomized controlled trial (RCT) of avelumab monotherapy versus avelumab plus pegylated liposomal doxorubicin and pegylated liposomal doxorubicin monotherapy among women with platinum resistant or refractory ovarian cancer. Neither immunotherapy arm outperformed the pegylated liposomal doxorubicin alone with regards to PFS or OS. However, among the patients whose tumors express PD-L1 and had CD8 positive cells, PFS and OS favored combination therapy. This group was not defined a priori but was part of prespecified biomarker analyses [53]. IMagyn500 was a phase III RCT comparing carboplatin, paclitaxel, bevacizumab, and atezolizumab versus carboplatin, paclitaxel, bevacizumab, and placebo in patients with newly diagnosed ovarian cancer. There was no difference in PFS or OS for the atezolizumab group compared to the placebo [54]. 

Additionally, several studies are ongoing. A phase I study is investigating the use of PF-07263689, a weakened pox virus vaccine that stimulates the immune system, alone and in combination with sasanlimab, a PD-1 monoclonal antibody, for various advanced solid cancers including ovarian cancer (NCT05061537). 

NRG-GY009 is a phase II/III trial comparing pegylated liposomal doxorubicin plus bevacizumab and/or atezolizumab among patients with platinum-resistant ovarian cancer (NCT02839707). Patients with recurrent or advanced dMMR or POLE mutated ovarian cancer were included in cohort F of the GARNET study, evaluating dostarlimab, an anti-PD-1 antibody. In an interim analysis, only two ovarian cancer patients were included in this basket trial: one with a partial response and one with stable disease [55,56]. Cohort G, evaluating platinum-resistant ovarian cancer without BRCA mutations, is still enrolling.

Checkpoint inhibitors have also been evaluated in combination with Poly (ADP-ribose) polymerase (*PARP*) inhibitors (PARPi) and anti-VEFG therapies. The rationale for these combinations is multifactorial but derives from TIME immunomodulation and the development of neoantigens to facilitate an immunogenic response. As an example, PARPi was observed to promote the accumulation of cytosolic DNA fragments, leading to activation of the cGAS-STING pathway and the production of interferons, thus inducing antitumor activity and augmenting the immune checkpoint blockade [57]. Moreover, PARP inhibition may increase PD-L1 expression in tumor cells, improving the efficacy of checkpoint inhibitors [58]. Multiple theories for additive or synergistic effects between PARPi and anti-VEGF therapies have been suggested. Anti-VEGF therapies induce tumor hypoxia, leading to the downregulation of BRCA 1/2 and RAD51 which may potentiate PARP inhibition [59,60]. Likewise, PARP1 knockout mice have decreased angiogenesis which may potentiate anti-VEGF therapies [61]. TOPACIO was a pooled phase I/II RCT assessing the efficacy of niraparib with pembrolizumab [62]. In the ovarian cancer cohort, there were 62 total patients with an objective response rate of 18% and a disease control rate of 65%, including 3 (5%) complete responses and 8 (13%) partial responses. The response rates were consistent regardless of BRCA or the homologous recombination deficiency (HRD) status. The DUO-O trial is an ongoing RCT comparing durvalumab in combination with chemotherapy and bevacizumab followed by maintenance bevacizumab with or without maintenance durvalumab and/or olaparib (NCT03737643). 

The efficacy of immunotherapy continues to be a challenge in ovarian cancer due to its particular combination of altered pathways and metabolism. One particular challenge is that in contrast to other cancer types, <2% of ovarian cancers exhibit mismatch repair deficiency (dMMR) [63]. The lack of a priori biomarker stratification may explain the limited efficacy of immunotherapy in clinical trials such as Javelin [46]. Additionally, several pathways frequently mutated in ovarian cancer may reduce the efficacy of immunotherapy. The PI3K-AKT-mTOR pathway is well known to drive tumor growth and proliferation but also has effects on the TIME. Akt reduces peroxisome proliferator-activated receptor gamma coactivator 1-alpha in TILs, leading to TIL exhaustion and decreased antitumoral immunity [64]. Though promising, tumor heterogeneity and a lack of reliable biomarkers has stymied the success of PI3K inhibition as a monotherapy [65]. Another hallmark of cancers, including ovarian cancer, is hypoxia due to inadequate vascularization in the setting of uncontrolled proliferation leading to activation of the hypoxia-inducible factor (HIF) pathway. HIF-1α promotes VEGF-A production, Treg and myeloid-derived suppressor cell recruitment, and shedding of NKG2D ligands, helping cancer cells escape from NK cells [66,67,68]. Combination therapies that target HIF-1α might improve the efficacy of immunotherapies. Similarly, JAK/STAT signaling has dual roles, both in tumorigenesis and immune evasion through decreasing interferon mediated tumor PD-L1 expression [69]. Overactive Wnt/β-catenin signaling, which may be driven by a number of mutations including the PTEN, KRAS, and BRAF genes, drives tumor progression and resistance to immunotherapy [65]. Wnt/β-catenin signaling inhibits dendritic cell recruitment and activation, favors polarization to Th2, and enhances Treg survival [70,71]. Together, these mutations are heterogeneous yet common in ovarian cancer and may contribute to resistance to immunotherapy. 

In addition to mutations in pathways that may contribute to a lack of efficacy of immunotherapy, metabolites and metabolism also play a role in ovarian cancer. The TIME is a unique environment that is nutrient poor and hypoxic due to the combination of uncontrolled tumor cell growth and poor vascularity. Tumor cells use a variety of mechanisms to allay these limitations. Tumors may use macropinocytosis, a process of taking up extracellular fluid in vesicles to acquire proteins, and entosis to engulf other cells and utilize their nutrients [72,73]. Through these mechanisms, tumor cells are able to outcompete other cells, especially for amino acids which are critical for the function of infiltrating immune cells, leading to their decreased fitness and survival capacity [74]. Immune cell deprivation of individual amino acids also leads to immunosuppression. For example, tumor cells use large amounts of glutamine and T cells in glutamine-deprived environments tend to differentiate into Tregs [75]. Similar findings have been found for arginine and tryptophan which, when at low concentrations, inhibit T cell function [76,77,78]. Together, these mechanisms contribute to a lack of immunotherapy efficacy and are among the many challenges to treating ovarian cancer. For a summary of relevant trials of checkpoint inhibitors in ovarian cancer, refer to Table 1. 

## 3. Endometrial Cancer

Endometrial cancer is the most common gynecologic malignancy in the United States, with 65,950 new cases and 12,550 deaths attributed to the disease projected for 2022. The incidence of endometrial cancer is on the rise and an accelerated mortality rate has been noted from 0.3% per year from 1997–2008 to 1.9% per year from 2008–2018 [1]. Worldwide, endometrial cancer is the second most common gynecologic cancer with 417,367 cases in 2020 but only 97,370 mortalities [2]. As bleeding is an early sign of disease, most women are diagnosed at early stages where the tumor is confined to the uterus with 5-year survival rates approaching 95%. However, survival rates for nearly 30% of women diagnosed at advanced stages are estimated at 18.4% for Stage IV disease and 69.8% for Stage III disease [79]. 

Classically, endometrial cancer has been classified as type I tumors and type II tumors. Type I tumors constitute about 80% of endometrial cancer diagnoses and often have favorable prognoses. They are characterized by exposure to excess and unopposed estrogen from both endogenous and exogenous sources. Type II endometrial cancers account for the remaining 20% of endometrial cancers and include non-endometrioid histologies, of which serous is the most common [80]. These cancers portend a poor prognosis and demonstrate more aggressive behavior with a high potential for extra-uterine spread. 

Combination chemotherapy with carboplatin and paclitaxel is the primary treatment option for patients with advanced or recurrent disease. The response rate to this regimen is 40-60%. However, few second-line therapy options exist and response rates are limited to about 20% or less [81,82,83].

In recent years, the TCGA has outlined four molecular subtypes of endometrial cancer including POLE ultramutated, microsatellite instability hypermutated, copy number low, and copy number high. The division into these subtypes has far-reaching implications for the way clinicians treat endometrial cancer, especially as it pertains to immunotherapy [84,85]. 

### 3.1. Tumor Immune Microenvironment in Endometrial Cancer and Association with Outcomes

As in ovarian cancer, both innate and adaptive immunity appear to play a role in tumorigenesis with specific subpopulations of infiltrating immune cells acting as prognostic indicators. Within the innate immune system, TAMs, as mentioned previously, are classified into M1 and M2 macrophages. While M1 macrophages are pro-inflammatory, M2 macrophages suppress the immune system and may promote tumor growth. Though the density of TAMs has demonstrated an association with prognosis in other solid tumors, the relationship in endometrial cancers is not well understood. In a study by Dun et al., a variety of hysterectomy specimens were stained with anti-CD68 (a marker for TAMs). Specimens were obtained from women undergoing hysterectomy for benign conditions, endometrial hyperplasia, Type I endometrial cancer, and Type II endometrial cancer. They found that the endometrial cancer specimens demonstrated higher TAM density in tumor and stromal tissue compared to benign specimens. While there was a trend of increasing TAM density with increasing FIGO grade, myometrial invasion, lymphovascular space invasion, lymph node metastasis and FIGO stage, the findings were not statistically significant. Additionally, no association was demonstrated between TAM density and clinical prognosis within endometrial cancer patients [86]. Further attempts have been made to delineate M1 and M2 subtypes in endometrial cancer. Kelly et al. found that while Type II endometrial cancers have nearly twice the density of CD68+ TAMs, the number of CD163+ M2 TAMs were relatively equal across Type I and II tumors. This suggests that Type II endometrial cancers may have a higher predominance of stromal pro-inflammatory M1 TAMs [87].

The adaptive immune system also appears to play an important role in endometrial cancer. In a study by Kondratiev et al., increased frequency of peritumoral infiltration by CD8+ cytotoxic T-cells in tumor cells correlated with increased overall survival [88]. Further characterization of the immune cell population making up these TILs demonstrated the importance of the balance between effector and suppressor responses in immune homeostasis. DeJong et al. examined three populations of TILs including CD8+ cytotoxic T-cells, regulatory T-cells (Treg) comprised of FoxP3+ T-lymphocytes, and memory CD45R0+ T-lymphocytes in tissue microarrays of 368 patients with endometrial cancer. They noted increased PFS and OS in patients with high rates of tumor infiltration by CD8+ T-lymphocytes. PFS was also associated with a high CD8+/FoxP3+ ratio and OS correlated with the presence of intra-tumoral CD45R0+ T-lymphocytes [89]. Tertiary lymphoid structures (TLSs) are quickly becoming an active area of investigation in the era of immunotherapy. TLSs are organized aggregates of immune cells in non-lymphoid tissues within the tumor immune microenvironment. These structures are enriched with CD4 and 8+ T cells, CD38+ plasma cells, and CD20+ B cells and facilitate a coordinated immune response. Qin et al. studied 104 patients with endometrial cancer and found that the presence of TLSs and CD20+ B cells were associated with improved PFS [90].

Similarly to ovarian cancer, endometrial cancer is a heterogeneous disease, with histology and TCGA classification having a significant effect or correlation with the TIME. Willvonseder et al. conducted a study of endometrioid endometrial cancer to evaluate the relationship and prognostic impact between TCGA-defined molecular groups based on immunohistochemistry, sequencing, and infiltration of TILs and Tregs. A high density of TILs was more common in high-grade tumors, POLE wildtype, and microsatellite stable (MSS) disease. Microsatellite instability (MSI) was associated with high-level TIL infiltration, and interestingly, Tregs were highly predictive of poor OS in p53 mutants [91]. Guo et al. used the TCGA as well, giving immune scores to each sample and using weighted gene co-expression network analysis to identify key genes correlating to immune scores. Their scoring system correlated strongly with tumor grade and histology. High immune scores were protective and they identified 11 key genes that correlated with immune cell infiltration and survival [92,93]. In a similar analysis, Li et al. identified four immune subtypes that exhibited specific molecular classifications and found that NK cells, dendritic cells, and CD8+ T cells were associated with survival [92]. Liang et al. extracted immune related genes from the TCGA and analyzed how three immune subtypes, clustered according to patterns of tumor mutational burden, immune response markers to chemotherapy, and immune checkpoint related genes, respond to immunotherapy. There was a high degree of heterogeneity but the second subtype identified was more sensitive to PD-L1 inhibitors. SIRPG and SLAMF1 were identified as immune-related biomarkers associated with favorable outcomes and a potential immune target for therapy [94]. Horeweg et al. sought to risk stratify early-stage endometrial cancer from the TCGA further based on intratumoral immune infiltration. They used a machine-learning, image-based algorithm to quantify CD8+ and CD103+ immune cells in endometrioid endometrial cancers from PORTEC-1 and PORTEC-2. Immune cell infiltration density varied substantially between cases and were clustered into three groups of high, intermediate, and low density. CD8+ cell density was the strongest predictor of recurrence, especially in p53 mutant tumors, but the effect was absent in DNA mismatch repair deficient cancers. Additionally, high immune cell infiltration was more common in POLE-mutant cases [95]. Given these results, it is clear that there is both a direct relationship between the TCGA type and the TIME, but as of yet, these results have not been evaluated prospectively or translated into clinical practice. 

It is clear that both the innate and adaptive immune system are important components of the tumor immune microenvironment in endometrial cancer. Most of what is known regarding specific immune subtypes in endometrial cancer is driven by research in T-cell populations. Further work is warranted to unravel the intricacies of the role of innate immunity subpopulations and B-cells to identify a wider diversity of potential therapeutic targets for endometrial cancer.

### 3.2. Therapeutic Targets in Endometrial Cancer

#### 3.2.1. Tumor Mutational Burden

In 2013, TCGA devised a new classification schema for endometrial cancer with the identification of four molecular subtypes: POLE ultra-mutated, microsatellite instability hypermutated, copy-number low, and copy-number high [84]. Tumors in the POLE hypermutated and microsatellite instability hypermutated (MSI-H) subgroups harbor defective DNA repair machinery leading to high rates of somatic mutations. These mutations increase the “neo-antigenic load” or tumor mutational burden (TMB) of a tumor and may make these groups of tumors particularly susceptible to immunotherapy [96,97]. 

The role of TMB, defined as the total number of mutations per megabase in the genomes of cancer cells, was investigated as a potential prognostic and predictive biomarker to immunotherapy in several cancer types. While the role of TMB as a predictive biomarker is still under investigation, high TMB found in certain subsets of endometrial cancer results in increased mutation-derived antigens that act as targets for the immune system in immunotherapy. Small studies in patients with melanoma, non-small cell lung cancer, and bladder cancer being treated with immune checkpoint inhibitors have demonstrated a positive association between tumors with a high tumor mutational burden, determined by whole exome sequencing, and response to CTLA-4 blockade and PD-1/PD-L1 inhibition independent of PD-L1 status [98,99,100,101]. Data on endometrial cancer have thus far been limited. However, in a post-hoc analysis from the phase I GARNET Trial (NCT02715284), endometrial cancer patients with high TMB (defined as ≥10 mutations/megabase on next generation sequencing) demonstrated higher overall response rates to the PD-L1 blockade by dostarlimab [102]. 

#### 3.2.2. Tumor Infiltrating Lymphocytes (TILs)

Further studies have demonstrated that the level of immune cell infiltration differs among molecular subtypes of endometrial cancer delineated by TCGA. Notably, tumors with a high tumor mutational burden, such as POLE and MSI-H endometrial cancers, have higher rates of TILs, as described above [103,104,105]. These data provide the rationale underlying the use of immunotherapy, specifically immune checkpoint inhibitors in endometrial cancer, with POLE ultramutated and MSI-H tumors deriving the most benefit due to the high amounts of pre-existing cytotoxic T-cells that can be induced to exert an anti-tumoral response [106]. 

#### 3.2.3. PD-L1

Within the immune cell milieu in endometrial cancer, a balance between effector and suppressor responses is maintained in large part by immune checkpoints that have been exploited as therapeutic targets for the treatment of endometrial cancer. PD1 is an inhibitory receptor that resides on the surface of various immune cells in both the innate and adaptive pathways including T-cells, NK cells, B-cells, dendritic cells, and monocytes. The binding of PD1 and its ligand PD-L1 results in the downregulation of cytotoxic T-cell activation and differentiation. The upregulation of PD-L1 by tumor cells contributes to the tumor’s ability to escape immune surveillance [107].

Endometrial cancer tends to exhibit high rates of PD1 and PD-L1 overexpression with studies demonstrating PD1 overexpression in 75% of cases and PD-L1 overexpression in 25–100% of cases [108,109]. When stratified by molecular subtypes, POLE and MSI-H endometrial cancers had significantly higher levels of expression of PD1/PD-L1 on peritumoral and intra-tumoral immune cells when compared to MSI stable tumors [97].

### 3.3. Checkpoint Inhibitors in Endometrial Cancer

Currently, there are limited therapeutic options for recurrent/advanced endometrial cancers that have progressed on platinum therapy. Immunotherapy, in particular using PD1/PD-L1 inhibitors, has shown promise, with pembrolizumab being the first immune checkpoint inhibitor explored in endometrial cancer. Since then, multiple trials have emerged investigating the role of PD1/PD-L1 inhibition as a monotherapy or in combination with other anti-neoplastic agents for the treatment of endometrial cancer. In the multi-cohort phase Ib trial, KEYNOTE-028 (NCT02054806), 24 endometrial cancer patients with locally advanced or metastatic PD-L1 positive (>1%) endometrial cancer were treated with pembrolizumab monotherapy. The ORR was 13%, with four patients achieving a PR (95% CI, 2.8–33.6%) and a disease control rate of 26.1% with an additional three patients with SD. The median PFS was reported as 1.8 months (95% CI, 1.6–2.7 months) and the median OS was not reached. Of the 24 patients included, 19 had tumor samples that could be evaluated for MSI status. Only one patient was found to have MSI-H status and this patient had a progressive disease [110]. In contrast, a phase II trial KEYNOTE-158 (NCT02628067) included 79 MSI-H endometrial cancer patients. The ORR was 48% (95% CI, 36.7–59.6%) and the disease control rate was 83.5% in this population. The median PFS was 13.1 months (95% CI, 4.3–34.4 months). The median OS was not reached [111]. 

Pembrolizumab has also been studied in combination with the tyrosine kinase inhibitor lenvatinib in a phase Ib/II trial KEYNOTE-146/Study 111. In total, 108 patients were included in this study and had a 24-week ORR of 36.2% (95% CI, 26.5–46.7%) in patients with MSI-S tumors compared to 63.6% (95% CI, 30.8–89.1%) in patients with MSI-H tumors. The disease control rate was 84% (95% CI, 75–90.8%) in MSI-S patients versus 90.9% (95% CI, 58.7–99.8%) in MSI patients. The median PFS was 7.4 months (95% CI, 5.0–7.6 months) in MSI-S patients and 18.9 months (95% CI, 13.5–25.9 months) in MSI-H patients. The median OS was 16.4 months in MSI-S patients (95% CI, 13.5–25.9 months) and was not reached in MSI-H patients [112].

Most recently, the ENGOT-EN-6-NSGO/GOG-3031/ RUBY (NCT03981796) and NRG-GY018 trials (NCT03914612) demonstrated practice-changing benefits of PD-1 inhibition in endometrial cancer. RUBY was a phase III RCT for patients with advanced stage or recurrent endometrial cancer treated with carboplatin, paclitaxel, and dostarlimab or carboplatin, paclitaxel, and placebo for 6 cycles followed by dostarlimab or placebo maintenance for up to 3 years [113]. Of the 494 patients who were randomized, 118 (23.9%) had dMMR or MSI-H tumors. In the dMMR-MSI-H population, PFS at 24 months was improved from 15.7% to 61.4% with dostarlimab (HR 0.28; 95% CI, 0.16–0.50; *p* < 0.001). This effect was less pronounced for the pMMR-MSS population, though a PFS benefit was still observed from 18.8% to 28.4% (HR 0.76; 95% CI, 0.59–0.98). In the overall group, OS at 24 months was improved from 56% to 71.3% with dostarlimab (HR 0.64; 95% CI, 0.46–0.87). There was not a significant improvement in OS for pMMR-MSS patients at the time of analysis, though the data are not yet mature. NRG-GY018 was a phase III RCT for patients with advanced or recurrent endometrial cancer [114]. The patients received carboplatin, paclitaxel with pembrolizumab, or carboplatin, paclitaxel with placebo for 6 cycles followed by up to 14 cycles of maintenance treatment with pembrolizumab or placebo. For the dMMR cohort, PFS at 12 months was 74% in the pembrolizumab group versus 38% in the placebo group (HR 0.30; 95% CI, 0.19–0.48; *p* < 0.001). In the pMMR cohort, the median PFS was 13.1 months for pembrolizumab and 8.7 months with the placebo (HR 0.54; 95% CI 0.41–0.71; *p* < 0.001). Final overall survival results are highly anticipated for both trials. For a summary of checkpoint inhibitors in endometrial cancer, refer to Table 2. 

## 4. Cervical Cancer

Cervical cancer is the most common gynecologic malignancy globally, with the highest burden of disease seen in resource poor settings without access to regular screening. In 2020, there were 604,127 new cases worldwide and 341,831 deaths [2]. With improved access to screening and human papilloma virus (HPV) vaccination, cervical cancer is less common and caught at earlier stages in resource rich countries. When disease is confined to the cervix, treatment can be curative with surgery. But, as the disease spreads locally or to the lymphatic system, chemoradiation is required and the disease becomes much more difficult to treat. Despite advances in prevention and treatment, over 13,000 new cases of cervical cancer are diagnosed annually in the US and 5-year survival rates for locally advanced and advanced cervical cancer remain at approximately 50%; therapy for recurrent and metastatic disease is limited [79,115]. 

### 4.1. Tumor Microenvironment in Cervical Cancer

An overwhelming majority of cervical cancer can be attributed to infection with oncogenic strains of HPV. High-risk strains of HPV such as HPV 16 and 18 encode genes for oncoproteins E6 and E7, which inhibit the actions of the important tumor suppressor genes p53 and Rb, respectively. These oncoproteins, in addition to their activity on tumor suppressor genes, also contribute to the ability of HPV to evade immune detection [116]. 

E7 oncoproteins act to downregulate pro-inflammatory immune signaling pathways in infected keratinocytes by blocking signaling via toll-like receptor 9 (TLR9) as well as the cGAS-STING pathway [117,118,119]. E6 and E7 have also been shown to interfere with interferon signaling and downregulate the NF-kB pathway as well as the production of Interleukin 1β, all contributing to the impairment of the host innate immune system, allowing for the persistence of HPV infection and tumorigenesis [120,121,122]. The effect of suppressing these inflammatory pathways is thought to lead to low levels of CCL20. CCL20 is a chemokine that attracts antigen-presenting cells such as Langerhans cells to sites of viral infection [123]. E7 also decreases E-cadherin expression which is important for adherence of Langerhans cells to the epidermis for viral antigen uptake [124]. 

E7 oncoproteins are further implicated in the downregulation of major histocompatibility complex (MHC) class I by binding to the MHC I promotor and recruiting histone deacetylases to repress gene expression [125]. Additionally, E5 viral proteins block the transport of MHC Class I from the Golgi apparatus and endoplasmic reticulum to the cell surface [126]. MHC Class I is required for antigen presentation and subsequent downstream activation of the adaptive immune response by cytotoxic T lymphocytes (CTLs). It is estimated that over 30% of HPV-associated cervical cancers have reduced MHC class I expression [127]. This downregulation of MHC class I has been implicated as a potential mechanism of resistance to anti-PD-1 and PD-L1 inhibitors and is a poor prognostic indicator in head and neck cancers [128]. The association with prognosis in cervical cancer is yet to be understood. 

In looking at TILs in cervical cancer, studies have shown higher levels of TILs in dysplastic cervical tissue compared to normal tissue, with a direct correlation between the level of TILs and increasing grade of cervical dysplasia [129,130,131]. From a prognostic standpoint, increased intratumoral and peritumoral density of both CD3+ and CD8+ T cells was shown to be associated with a decreased risk of relapse, with peritumoral CD3+ T cells having the strongest association [132]. CD3+ is an antigen that is present on all T cells, including helper, regulatory, and cytotoxic T cells. The findings in this study seem to suggest that, while cytotoxic CD8+ T cells are the main effectors of the direct killing of intracellular pathogens, a variety of immune cell types are necessary to exert an immunologic response. 

Additional studies have indicated that a higher level of CD8+ T cells may predict a better response to treatment in patients with squamous cell carcinoma of the cervix. A study by Martins et al. evaluated samples from 21 patients receiving treatment with concurrent chemoradiation and found that patients who responded to therapy had a more inflammatory tumor microenvironment, designated by higher numbers of CD8+ TILs compared to non-responders [133]. In another study of 71 patients with adenocarcinoma of the cervix undergoing definitive RT with or without concurrent chemotherapy, the presence of intratumoral CD8+ TILs had significantly better OS compared to those without infiltrating CD8+ T cells following treatment [134].

FoxP3+ TILs are Treg cells that inhibit the function of effector TILs such as CD8+ cytotoxic T cells. In cervical cancer, increased levels of FoxP3+ cells detected by immunohistochemistry are associated with increased HPV viral load, higher FIGO stage, increased recurrence rate, and decreased 15-year OS [135].

Less studied is the role of the TIME in HPV-negative cervical cancers due to their relative rarity. Evans et al. utilized the TCGA dataset to compare the TIME of HPV16+, HPV18+, and HPV-negative cervical cancer, finding greatly reduced TIL and reduced markers for B, T, and NK cells. Interestingly, they did find that HPV-negative cervical cancer had higher levels of potential neoantigens. Unfortunately, the HPV-negative samples were too rare to correlate findings with survival in this group [136]. Other data are limited, but data from other disease sites such as oropharyngeal cancer support the concept of a cold TIME in HPV-negative cancers relative to HPV-positive counterparts [137,138]. 

The role of infection with high-risk strains of HPV in the pathogenesis of cervical cancer provides a strong rationale for the use of immunotherapy in this disease site. Ongoing therapeutic options include a checkpoint blockade with the use of PD-1/PD-L1 inhibitors but also CTLA-4 targeting agents. Beyond checkpoint blockade, therapeutic vaccines represent an exciting strategy currently under investigation. 

### 4.2. Therapeutic Targets in Cervical Cancer

#### 4.2.1. ADXS-HPV

One especially promising option for therapeutic vaccines includes bacterial vector-based vaccines. Axalimogene filolisbac (ADXS-HPV) uses attenuated *Listeria monocytogenes* as the bacterial vector for a fusion HPV E7 protein and stimulates a CD4+ and CD8+ T cell adaptive response. It has been studied in a variety of HPV-associated cancers including head and neck cancer [135,139,140] and anal cancer [141,142] but has been evaluated most extensively in cervical cancer. Huh et al. studied ADXS-HPV in a phase II clinical trial that included 50 patients with advanced cervical cancer. The ORR in this study was 6% with a DCR of 16%. Twelve-month OS was 38% with a median OS of 6.1 months (95% CI, 4.3–12.1). The median PFS was reported as 2.8mo (95% CI, 2.6–3.0) [143].

#### 4.2.2. ACT

Adoptive T-cell therapy (ACT) is also an area of ongoing research. In ACT, a patient’s own tumor-reactive T cells are isolated and expanded. These T-cells have already been primed against HPV-oncoproteins of the patient’s tumor and are given as a systemic infusion. In 2019, a phase II trial by Stevanović et al. demonstrated an ORR in 5/18 (28%) of cervical cancer patients, 2 of which were durable complete responses lasting >50 months [144]. 

#### 4.2.3. PD-1/PD-L1

Several studies have demonstrated a positive association between HPV positivity and PD-L1/PD-1 expression. Mechanistically, HPV E5, E6, and E7 oncogenes act via multiple signaling pathways to induce expression of PD-L1 and PD-1 leading to suppression of the adaptive immune response allowing for progression of disease [145]. Both PD-L1 and PD-1 are expressed at relatively high levels in cervical cancer. While PD-L1 is expressed on the surface of cervical tumor cells, antigen presenting cells and TILs, PD-1 is expressed in T cells in the stroma of cervical tumors. Several studies have evaluated rates of PD-L1 and PD-1 expression in cervical cancers with ranges reported between 34.4–96% [146,147,148] and 46.97–60.82% [149,150], respectively, making cervical cancer an attractive candidate for checkpoint inhibitors. 

### 4.3. Checkpoint Inhibitors in Cervical Cancer

Since 2015, there have been several clinical trials evaluating the efficacy of checkpoint inhibitors in cervical cancer both in the upfront as well as recurrent setting. Currently, pembrolizumab has FDA approval in combination with chemotherapy with or without bevacizumab for patients with advanced and recurrent cervical cancer with PD-L1 tumor expression (CPS ≥ 1.) It is additionally approved as a single agent for PD-L1 expressing recurrent/metastatic cervical cancer in patients who have disease progression on or after receiving chemotherapy. The approval for these indications is based on results from the phase III trial, KEYNOTE-826 (NCT03635567), which studied pembrolizumab in combination with chemotherapy (paclitaxel/cisplatin or paclitaxel/carboplatin) with or without bevacizumab compared to the placebo plus chemotherapy with or without bevacizumab in the treatment of persistent/recurrent/advanced cervical cancer. The study included 548 patients with a PD-L1 CPS ≥1 and noted longer PFS in the pembrolizumab group with median PFS reported as 10.4 months vs. 8.2 months in the placebo group (HR 0.62; 95% CI, 0.50–0.77; *p* < 0.001). OS in the pembrolizumab group at 24 months was 53% vs. 41.7% in the placebo group and the median OS was not reached [151].

With pembrolizumab approved, several other drugs targeting the PD-L1/PD-1 axis are currently under investigation. In the most recently published EMPOWER-Cervical 1 trial (NCT03257267), cemiplimab, a PD-1 blocking agent used in the treatment of lung and skin cancers, was compared to the investigator’s choice chemotherapy in patients with disease progression after first-line platinum-containing chemotherapy. Patients were enrolled regardless of their PD-L1 status. The results demonstrated the median OS in the cemiplimab group as 12.0 months vs. 8.5 months (HR 0.69; 95% CI, 0.56–0.84; *p* < 0.001). PFS was longer in the cemiplimab group compared to the chemotherapy group. The ORR was reported as 16.4% (95% CI, 12.5–21.1%) in the cemiplimab group and 6.3% (95% CI, 3.8–9.6%) in the chemotherapy group. When looking at patients with PD-L1 CPS ≥ 1, the ORR was 18% (95% CI, 11–28%). Notably, for patients with CPS ≤ 1, the ORR was 11% (95% CI, 4–25%) [152]. 

Other compounds under investigation include CTLA-4 inhibitors. CTLA-4 is a T-cell surface receptor expressed on regulatory T cells and binds to CD80 and CD86 as ligands to dampen the immune response. By blocking this interaction of CTLA-4 and CD80/CD86, CTLA-4 inhibitors aim to allow activation of the immune system [153]. Currently, CTLA-4 inhibitors such as ipilimumab are being evaluated as a monotherapy and in combination with other targeted agents as well as with standard chemotherapies. The combination of ipilimumab and nivolumab was studied in the phase I/II CheckMate 358 trial (NCT02488759). Nineteen cervical cancer patients with recurrent/metastatic disease were enrolled in this trial. The reported ORR was 26.3% and the median OS was 21.9 mo (95% CI, 15.1–NR). The median PFS was 5.1 mo (95% CI: 1.9–9.1) [154]. O’Malley et al. studied a similar combination of PD-1 and CTLA-4 blockade with the drugs balstilimab and zalifrelimab. In this phase II trial (NCT03495882), 155 women with recurrent/metastatic cervical cancer were included: the ORR was 25.6% including 10 CRs and 22 PRs. The median PFS was reported as 2.7 mo (95% CI, 1.5–3.7) and the median OS was 12.8 mo (95% CI 8.8–17.6). Patients were enrolled regardless of their PD-L1 status but on subgroup analysis, the PD-L1 positive group had a higher ORR of 32.8% versus 9.1% for the PD-L1 negative group [155]. 

For a summary of checkpoint inhibitors in cervical cancer, refer to Table 3. 

## 5. Vulvar and Vaginal Cancer

Vulvar cancer is a rare disease, constituting 3–5% of gynecologic malignancies with an annual incidence of 1–2 per 100,000 women [156]. Vaginal cancer accounts for 2% of gynecologic malignancy and affects 1 in 100,000 women [157]. Vulvar cancer is rare globally with only 45,240 cases and 17,427 deaths in 2020, while vaginal cancer is even rarer, affecting 17,908 new patients in 2020 with 7995 deaths [2]. Both diseases are challenging to treat due to a propensity to recur multiple times and the fact that their rarity precludes effective clinical trial enrollment. Approximately 80% of vulvar squamous cell carcinoma is related to TP53 mutations and tend to arise from vulvar intraepithelial neoplasia, a differentiated type, often in the background of lichen sclerosis. HPV-associated vulvar squamous cell carcinoma (VSSC) is most often caused by HPV 16 and rises from high-grade squamous intraepithelial lesions (HSIL). 

### 5.1. Tumor Immune Microenvironment of Vulvar and Vaginal Cancer

The immune microenvironment of vulvar cancer has not been thoroughly studied and there is little to no published work regarding the TIME in vaginal cancer. In vulvar HSIL, progression is associated with increased CD14+ CD33- CD163+ mature (activated) M2 macrophages, which correlates with decreased CD8 T cells, increased Tregs, and decreased recurrence-free survival [158]. Antigen-presenting cells such as Langerhans cells (LCs) and dendritic cells (DCs) have been shown to have decreased infiltration into VSCC and lichen sclerosis [159,160]. Metastatic involvement of tumor draining lymph nodes has been associated with immune suppressive features including hampered DC activation, increased Treg CTLA-4 expression, and elevated PD-1 expression [161]. For HPV-induced lesions, a strong influx of CD4+ and CD8+ T cells is strongly associated with regression, while a lack of HPV-specific interferon-producing T cells is associated with HPV-induced cancer [162]. Activated cytotoxic CD56+ GrB+ intraepithelial lymphocytes correlate with longer survival [163]. The impact of Tregs is less well defined with mixed results. However, the limited studies published have not investigated the role of stromal or epithelial Tregs [164].

### 5.2. Immune Checkpoint Inhibitors in Vulvar and Vaginal Cancer

Immunotherapy has been evaluated in small numbers of patients with VSSC. In Keynote-028 (NCT02054806), 18 patients with VSCC that had prior disease progression on chemotherapy were included and they had an ORR of 6% with a median OS of 3.9 months [165]. In Keynote 158 (NCT02628067) for 101 patients with VSCC, the ORR was 10.9% among all patients. Interestingly, the ORR for the 84 patients with PD-L1 positive tumors was 9.5% and 28.6% among the 7 patients with PD-L1 negative tumors. For patients with a response, the median duration of response (DOR) was 20.4 months. The median PFS was 2.1 months and the median OS was 6.2 months [166]. In Checkmate 358 (NCT02488759), a phase I/II trial of nivolumab in recurrent or metastatic cervical, vaginal, or vulvar carcinoma, there were five vaginal/vulvar SCC patients. The ORR was 20% with a DOR of 5 months [154]. Additionally, a single arm phase II clinical trial for unresectable, incompletely resected, recurrent, or metastatic vulvar squamous cell carcinoma treated with cisplatin, pembrolizumab, and radiotherapy is ongoing (NCT04430699). Given these trial results and extrapolation from cervical cancer data, pembrolizumab is FDA approved and current NCCN guidelines endorse pembrolizumab for second-line therapy for TMB-H, PD-L1 positive, or MSI-high/dMMR tumors [167]. Nivolumab is also a therapeutic option for HPV-related advanced or recurrent/metastatic vulvar cancer [167]. For a summary of clinical trials of checkpoint inhibitors in vulvar and vaginal cancer, refer to Table 4.

## 6. Conclusions

Our understanding of the tumor immune microenvironment and its role in the development, progression, and treatment response of gynecologic malignancies continues to expand. With that expansion comes an increasing focus on targeted therapeutic options that aim to exploit the underpinnings of a host’s innate and adaptive immune response to exert cytotoxic effects on tumor cells. Whether as monotherapy or in combination with traditional treatment modalities, immunotherapy has clearly shifted the landscape of the management of gynecologic malignancies, ushering in a new era of precision medicine. As we accrue more data regarding the safety and efficacy of these drugs, it will become increasingly important to refine our understanding of the biomarkers that may be predictors of a response in order to better identify patients that may benefit from immunotherapy. 

## Figures and Tables

**Table 1:** Checkpoint inhibitors in ovarian cancer.

Trial	Population	Groups	N	Response Rate	Median PFS	Median OS	HR (95% CI)	Other
Keynote 100 Phase II NCT02674061	Advanced/ Recurrent	Pembro 1-3 prior lines 4-6 prior lines	285 91	ORR 7.4% ORR 9.9%	2.1 mo 2.1 mo	NR 17.6 mo		CPS < 1 ORR 5.0% CPS ≥ 1 ORR 10.2% CPS > 10 ORR 17.1%
NRG GY003 Phase II NCT02498600	Persistent/ Recurrent	Nivo Ipi + Nivo	49 51	ORR 12.2% ORR 31.4%	2.0 mo 3.9 mo	21.8 mo 28.1 mo	PFS 0.53 (0.34–0.82) OS 0.79 (0.44–1.42)	
Javelin Ovarian 100 Phase III NCT02718417	Stage III/IV Up front	TC TC + mAve TC + Ave+ mAve	335 332 331	ORR 27.8% ORR 25.9% ORR 31.1%	NR 16.8 mo 18.1 mo		- PFS 1.43 (1.05–1.95) PFS 1.14 (0.83–1.56)	
Javelin 200 Phase II NCT02580058	Platinum Resistant/ Refractory	PLD Ave PLD + Ave	190 188 188	ORR 4.2% ORR 3.7% ORR 13.3%	3.5 mo 1.9 mo 3.7 mo	13.1 mo 11.8 mo 15.7 mo	- OS 1.14 (0.95–1.58) OS 0.89 (0.74–1.24)	
IMagyn500 Phase III NCT03038100	Stage III/IV Up front	TC + Bev TC + Bev + Atez			18.4 mo 19.5 mo	NR NR	PFS 0.92 (0.79–1.07) OS 0.96 (0.74–1.26)	
TOPACIO Phase I/II NCT02657889	Platinum Resistant	Niraparib + Pembro	62	ORR 18%				
NRG-GY009 Phase II/III NCT02839707	Recurrent Platinum Resistant	PLD + Bev PLD + Bev Atez						Active, not recruiting
DUO-O Phase III NCT03737643	Stage III/IV Up front	TC + Bev TC + Bev + Durv TC + Bev + Durv + Olaparib						Active, not recruiting
Garnet Phase I NCT02715284	Advanced/ Recurrent	Dostarlimab: Cohort F dMMR/MSI-H Cohort G PROC, without BRCA mutation	2	ORR 50%				Active, recruiting

Pembro, pembrolizumab; Nivo, nivolumab; Ipi, ipilimumab; TC, carboplatin paclitaxel; mAve, maintenance avelumab; Ave, avelumab; Bev, bevacizumab; Atez, atezolizumab; PLD, pegylated liposomal doxorubicin; Durv, durvalumab; PROC, platinum resistant ovarian cancer; HR, hazard ratio; mo, months; NR, not reached; OS, overall survival; PFS, progression-free survival.

**Table 2:** Checkpoint inhibitors in endometrial cancer.

Trial	Population	Groups	N	ORR	Median PFS (Months)	Median OS (Months)
GARNET Phase I NCT0271528	Advanced/Recurrent	Dostarlimab	104	dMMR/MSI-H: 43.5% MMRp/MSS: 14.1%		
KEYNOTE-028 Phase Ib NCT0205480	Advanced/Recurrent PD-L1+	Pembro	24	13%	1.8 (95% CI, 1.6–2.7)	NR
KEYNOTE-158 Phase II NCT0262806	Advanced/Recurrent dMMR/MSI-H	Pembro	79	48%	13.1 (95% CI, 4.3–34.4)	NR
KEYNOTE-146/Study 111 Phase Ib/II NCT0250109	Advanced/Recurrent	Pembro/Lenvatinib	108	dMMR/MSI-H: 63.6% MMRp/MSS: 36.2%	18.9 (95% CI, 13.5–25.9) 7.3 (95% CI, 5.2–8.7)	NR 16.4 (95% CI, 13.5–25.9)
ENGOT-EN-6-NSGO/ GOG-3031/ RUBY Phase III NCT03981796	Advanced/Recurrent	TC + Dostarlimab TC + Placebo	24 249		24–month PFS: 36.1% (95% CI, 13.0–23.9) 24–month OS: 18.1% (95% CI, 13.0–23.9)	24–month PFS: 71.3% (95% CI, 64.5–77.1) 24–month OS: 56.0% (95% CI, 48.9–62.5)
NRG-GY018 Phase III NCT03914612	Advanced/Recurrent	TC+ Pembro	407		dMMR/MSI–H: NR MMRp/MSS: 13.1 (95% CI, 10.5–18.8)	NR
		TC + Placebo	408		dMMR/MSI-H: 7.6 (95% CI, 6.4–9.9) MMRp/MSS: 8.7 (95% CI, 8.4–10.7)	

Pembro, pembrolizumab; TC, carboplatin paclitaxel; HR, hazard ratio; mo, months; NR, not reached; OS, overall survival; PFS, progression-free survival; dMMR, Mismatch Repair Deficient; MSI-H microsatellite high; MSS- Microsatellite stable.

**Table 3:** Checkpoint inhibitors in cervical cancer.

Trial	Population	Groups	N	ORR	Median PFS (Months)	Median OS (Months)
KEYNOTE-826 Phase III NCT03635567	Advanced/Recurrent	Cis + Pembro +/− Bev Cis + Placebo +/− Bev	308 309	65.9% 50.8%	10.4 (95% CI, 9.1–12.1) 8.2 (95% CI, 6.4–8.4)	24 months OS: 50.4% (95% CI, 43.8–56.6) 24 months OS: 40.4% (95% CI, 34.0–46.6)
EMPOWER-Cervical 1/ GOG-2016/ ENGOT-cx9 Phase III NCT03257267	Recurrent	Cemiplimab Investigator’s choice single agent chemotherapy	304 304	16.4% 6.3%	2.8 (95% CI, 2.6–3.9) 2.9 (95% CI, 2.7 –3.4)	12 (95% CI, 10.3–13.5) 8.5 (95% CI 7.5–9.5)
Checkmate-358 Phase I/II NCT02488759	Advanced/Recurrent	Ipilimumab/Nivolumab	19	26.3%	5.1 months (95% CI, 1.9–9.1)	21.9 (95% CI, 14.1–NR)
O’Malley et al. Phase II NCT03495882	Advance/recurrent	Balstilimab/Zalifrelimab	155	25.6%	2.7 months (95% CI, 1.5–3.7)	12.8 (95% CI 8.8–17.6)

Cis Cisplatin; Pembro Pembrolizumab; Bev Bevacizumab; OS, overall survival; PFS, progression-free survival; ORR, overall response rate.

**Table 4:** Checkpoint inhibitors in vulvar/vaginal cancer.

Trial	Population	Groups	N	Response Rate	Median PFS	Median OS	Other
Keynote-028 Phase IB NCT02054806	Advanced/ Metastatic	Pembrolizumab	18	ORR 6%	3.1 months	3.8 months	
Keynote 158 Phase II NCT02628067	Advanced	Pembrolizumab	101	ORR 10.9%	2.1 months	6.2 months	
Checkmate 358 Phase I/II NCT02488759	Advanced/ Metastatic/ Recurrent	Nivolumab	5	ORR 20%			
Phase II NCT04430699	Advanced/ Metastatic/ Recurrent	Cisplatin + Pembrolizumab+ Radiation					Recruiting

HR, hazard ratio; mo, months; OS, overall survival; PFS, progression-free survival.
